# Abaloparatide is an Effective Treatment Option for Postmenopausal Osteoporosis: Review of the Number Needed to Treat Compared with Teriparatide

**DOI:** 10.1007/s00223-018-0450-0

**Published:** 2018-06-27

**Authors:** Jean-Yves Reginster, Gary Hattersley, Gregory C. Williams, Ming-yi Hu, Lorraine A. Fitzpatrick, E. Michael Lewiecki

**Affiliations:** 1 Université de Liège, Place du 20-Août, 7, 4000 Liège, Belgium; 2 Radius Health, Inc., 950 Winter St, Waltham, MA 02451 USA; 3 New Mexico Clinical Research & Osteoporosis Center, Inc., 300 Oak St, SE, Albuquerque, NM 87106 USA

**Keywords:** Postmenopausal osteoporosis, Abaloparatide, Number needed to treat, ACTIVE trial, Fracture risk reduction

## Abstract

Abaloparatide (ABL) is a 34-amino acid peptide designed to be a selective activator of the parathyroid hormone receptor type 1 signaling pathway. In the Abaloparatide Comparator Trial In Vertebral Endpoints (ACTIVE), subcutaneous ABL reduced the risk of new vertebral, nonvertebral, clinical, and major osteoporotic fracture compared with placebo and of major osteoporotic fracture compared with teriparatide. To further evaluate the effectiveness of ABL, we calculated the number needed to treat (NNT) to prevent one fracture using ACTIVE data. To estimate the potential effectiveness of ABL in populations at higher fracture risk than in ACTIVE, we calculated NNT for vertebral fracture using reference populations from historical placebo-controlled trials, assuming an 86% relative risk reduction in vertebral fracture with ABL treatment as observed in ACTIVE. NNT was calculated as the reciprocal of the absolute risk reduction in ACTIVE. The projected NNT for ABL in other populations was calculated based on incidence rate (IR) for vertebral fractures in the placebo arms of the FREEDOM (placebo IR 7.2%), FIT-1 (placebo IR 15.0%), and FIT-2 (placebo IR 3.8%) trials. NNT for ABL in ACTIVE was 28 for vertebral, 55 for nonvertebral, 37 for clinical, and 34 for major osteoporotic fracture. NNT for these fracture types for teriparatide in ACTIVE were 30, 92, 59, and 75, respectively. Using placebo IRs from FREEDOM, FIT-1, and FIT-2, projected NNTs for vertebral fracture with ABL were 17, 8, and 31. These data are useful for further evaluating ABL for the treatment of osteoporosis in postmenopausal women.

## Introduction

Abaloparatide (ABL) is a 34-amino acid peptide designed to be a selective activator of the parathyroid hormone receptor type 1 (PTHR1) signaling pathway. ABL binds selectively to the RG versus R^0^ conformation of PTHR1 [1], consistent with a net anabolic effect in contrast with PTH [2]. Preclinical and clinical studies of ABL resulted in increases in bone mass and bone mineral density (BMD), restoration of bone microarchitecture, and increased bone strength with no adverse effects on bone quality and no apparent concerns regarding bone safety [3–8].

In the 18-month phase 3 Abaloparatide Comparator Trial In Vertebral Endpoints (ACTIVE, NCT01343004), treatment with subcutaneously administered ABL for 18 months significantly increased BMD and decreased the risk of vertebral, nonvertebral, clinical, and major osteoporotic fractures compared with placebo. ACTIVE included an open-label teriparatide (TPTD) arm, and ABL significantly increased BMD at nonvertebral sites and significantly decreased the risk of major osteoporotic fractures compared with TPTD [5].

To further elucidate the effectiveness of ABL, we evaluated the number needed to treat (NNT) to prevent one fracture (vertebral, nonvertebral, clinical, and major osteoporotic) using data from ACTIVE. To understand the potential effectiveness of ABL in patient populations at greater risk of fracture and with varying treatment durations, we also estimated the NNT for ABL using reference populations from historical placebo-controlled trials of treatments for postmenopausal women with osteoporosis.

## Methods

### Study Patients and Treatments

ACTIVE was a multicenter, multinational study that enrolled postmenopausal women (aged 49–86 years) who had osteoporosis. Eligibility criteria have been described in detail [5]. Briefly, osteoporosis was defined as having prior radiographic evidence of vertebral fracture or low-trauma nonvertebral fracture within 5 years of study enrollment and a T-score ≤ −2.5 and > −5.0 at the lumbar spine or femoral neck for women aged ≤ 65 years or a T-score ≤ −2.0 at those same sites for women aged > 65 years. For women older than 65 years, there was no prior fracture requirement if the lumbar spine or femoral neck T-score was ≤ −3.0 and > −5.0. Eligible women were randomized 1:1:1 to receive either blinded ABL 80 µg/d or matching placebo or open-label subcutaneous injections of TPTD 20 µg/d for 18 months. The intent-to-treat population included 2463 patients, all of whom received supplementary calcium and vitamin D.

### NNT Calculations

NNT to prevent one additional fracture (vertebral, nonvertebral, clinical, major osteoporotic) was calculated as the reciprocal of the absolute risk reduction (ARR) between the ABL and placebo groups and between the TPTD and placebo groups [9].

To project the potential effectiveness of ABL in other patient populations with different baseline levels of fracture risk than that in ACTIVE, we estimated the NNT for ABL in such populations by applying the 86% relative risk reduction (RRR) of vertebral fractures observed with ABL compared with placebo in ACTIVE to placebo incidence rates (IR) reported in historical reference populations. These reference populations included the Fracture Reduction Evaluation of Denosumab in Osteoporosis Every 6 Months trial (FREEDOM; denosumab vs. placebo for 3 years; placebo IR = 7.2%) [10]; the Fracture Intervention Trial-1 (FIT-1; alendronate vs. placebo for 3 years; placebo IR = 15.0%) [11]; and the Fracture Intervention Trial-2 (FIT-2; alendronate vs. placebo for 4 years; placebo IR = 3.8%) [12].

## Results

**Fig. 1:**
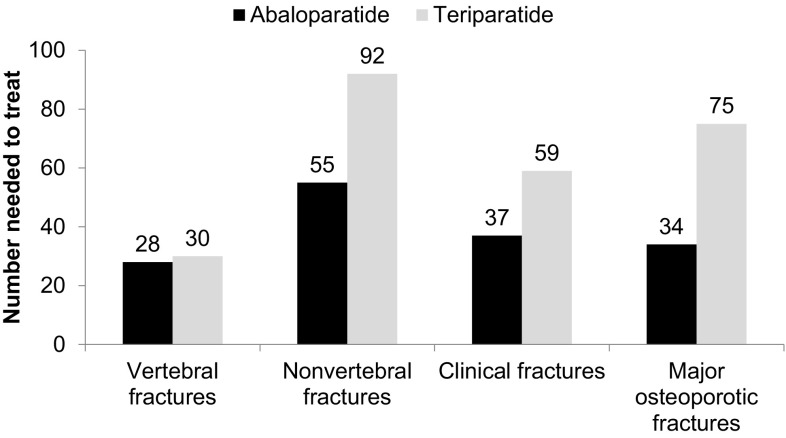
Number needed to treat, by fracture type, after 18 months of treatment with abaloparatide or teriparatide in ACTIVE

Fracture incidence rates for placebo, ABL, and TPTD following 18 months of treatment on ACTIVE [5] are displayed in Table 1. The NNT values after 18 months of treatment with ABL or TPTD in ACTIVE are presented in Fig. 1. The NNT for new vertebral fractures was 28 for ABL and 30 for TPTD. The NNT for nonvertebral, clinical, and major osteoporotic fractures were 55 and 92, 37 and 59, and 34 and 75, for ABL and TPTD, respectively.

**Table 1:** Fracture results after 18 months of treatment in ACTIVE. Reproduced with permission from Miller et al. [5]. Copyright©(2016) American Medical Association. All rights reserved

	Incidence *n* (%)^a^			ABL versus placebo			TPTD versus placebo			ABL versus TPTD		
	Placebo (*n* = 821)	ABL (*n* = 824)	TPTD (*n* = 818)	RD (95% CI)	HR^b^ (95% CI)	*P* value^c^	RD (95% CI)	HR^b^ (95% CI)	*P* value^c^	RD (95% CI)	HR^b^ (95% CI)	*P* value^c^
New vertebral fracture	30 (4.2)	4 (0.6)	6 (0.8)	−3.64 (−5.42 to −2.10)	RR, 0.14 (0.05 to 0.39)	< 0.001	−3.38 (−5.18 to −1.80)	RR, 0.20 (0.08 to 0.47)	< 0.001			
Nonvertebral fracture	33 (4.7)	18 (2.7)	24 (3.3)	−2.01 (−4.02 to −0.00)	0.57 (0.32 to 1.00)	0.049	−1.46 (−3.50 to 0.58)	0.72 (0.42 to 1.22)	0.22	−0.55 (−2.34 to 1.24)	0.79 (0.43 to 1.45)	0.44
Major osteoporotic fracture	34 (6.2)	10 (1.5)	23 (3.1)	−4.73 (−8.07 to −1.40)	0.30 (0.15 to 0.61)	< 0.001	−3.09 (−6.53 to 0.36)	0.67 (0.39 to 1.14)	0.14	−1.65 (−3.18 to −0.11)	0.45 (0.21 to 0.95)	0.03
Clinical fracture	49 (8.3)	27 (4.0)	35 (4.8)	−4.24 (−7.93 to −0.54)	0.57 (0.35 to 0.91)	0.02	−3.51 (−7.22 to 0.21)	0.71 (0.46 to 1.09)	0.11	−0.73 (−2.89 to −1.43)	0.81 (0.49 to 1.33)	0.40

*ABL* abaloparatide, *HR* hazard ratio, *RD* risk difference, *RR* relative risk, *TPTD*, teriparatide

^a^Percentage of new vertebral fractures was calculated using the modified intent-to-treat population, which included all patients with both pretreatment and postbaseline spine X-rays (placebo, *n* = 711; abaloparatide, *n* = 690; teriparatide, *n* = 717). Percentage of nonvertebral, major osteoporotic, and clinical fractures are cumulative Kaplan–Meier estimates using the intent-to-treat population at 19 months (18 months of treatment plus 1 month of follow-up)

^b^Values for new vertebral fractures are reported as relative risks; values for nonvertebral, major osteoporotic, and clinical fractures are reported as hazard ratios

^c^*P* values for new vertebral fractures were derived using the Fisher exact test. *P* values for nonvertebral, major osteoporotic, and clinical fractures were calculated using the log-rank test

**Fig. 2:**
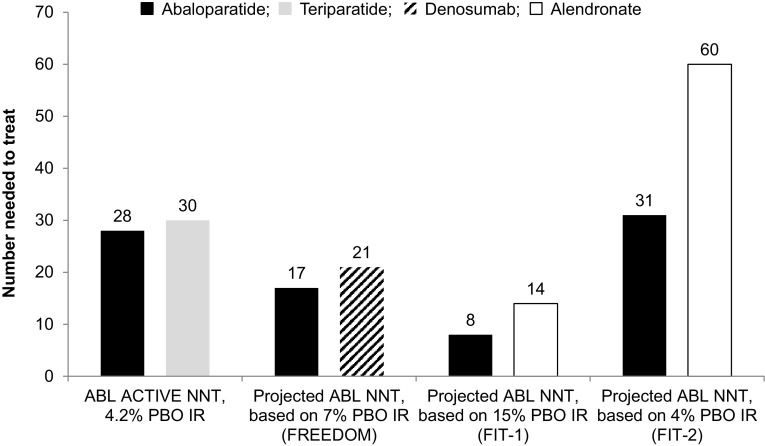
Projected number needed to treat with ABL based on incidence rates reported in populations with varying placebo-group incidence rates of new vertebral fracture. NNTs included for denosumab and for alendronate are as reported in FREEDOM [10], FIT-1 [11], and FIT-2 [12]. *ABL* abaloparatide, *IR* incidence rate, *NNT* number needed to treat, *PBO* placebo.

Projected NNTs for vertebral fractures based on placebo IRs reported in historical study populations are described in Fig. 2. Applying an 86% RRR in vertebral fracture to a placebo population with a 4% IR of new vertebral fracture, as seen in FIT-2 [12], yielded a projected NNT of 31 for ABL, while applying an 86% RRR to a placebo population with a 7% IR, as seen in FREEDOM [10], yielded a projected NNT of 17 for ABL. Finally, applying an 86% RRR in vertebral fracture to a placebo population with a 15% IR of new vertebral fracture, as seen in FIT-1 [11], yielded a projected NNT of 8 for ABL.

## Discussion

The NNT is the reciprocal of the ARR and is expressed as a single value, representing the number of patients who must be treated to prevent one event of interest [9]. As discussed by Cranney et al., NNT is one way of describing the absolute impact of treatment [13], and the NNT for osteoporosis therapy can be considered by clinicians when making recommendations to patients and when making comparisons of cost effectiveness.

During ACTIVE, ABL reduced the risk of vertebral, nonvertebral, major osteoporotic, and clinical fractures compared with placebo and reduced the risk of major osteoporotic fractures compared with TPTD [5]. There was a consistent fracture risk reduction across a variety of baseline risk factors, including age groups (< 65 vs. 65 to < 75 vs. ≥ 75 years) in ACTIVE [14, 15].

The analyses described here show that following 18 months of treatment in ACTIVE, the NNT for ABL versus placebo was lower than that of TPTD versus placebo for multiple fracture endpoints. The NNT for ABL and TPTD in ACTIVE were similar for new vertebral fractures: 28 for ABL; 30 for TPTD. Additional fracture types beyond new vertebral fractures of course contribute significantly to disease burden and are of clinical interest when evaluating treatments for osteoporosis [16]. In this analysis, there was considerable divergence in the NNT between ABL and TPTD for other fracture types, with ABL NNT ranging from 37% less for clinical fracture (37 for ABL vs. 59 for TPTD) to 55% less for major osteoporotic fracture (34 for ABL vs. 75 for TPTD).

The observed treatment differences between ABL and TPTD may be explained at least in part by the underlying physiological differences, and subsequent different binding profiles, of TPTD, a PTH analog, and ABL, a PTHrP analog [2, 17]. PTH binds with greater affinity and stability to the R^0^ conformation of the PTH1R than does PTHrP. Conversely, PTHrP has greater binding affinity for the RG conformation of the receptor, which results in more transient receptor signaling. Thus, despite PTH and PTHrP analogs binding to the same PTH1R, the different signaling pathways they affect appear to induce different biological responses [2].

The incidence of new morphometric vertebral fractures in the placebo group in ACTIVE was 4.2%, which was lower than the IRs of new vertebral fractures among placebo groups from several other trials of drugs to treat postmenopausal osteoporosis. Contemporary ethical concerns around assigning patients with severe disease to a placebo group led the ACTIVE investigators to enroll patients with lower risk of fractures than in many earlier clinical trials of other antifracture medications. To help understand the potential effects of ABL in populations that may have greater risk of new vertebral fracture than the 4.2% placebo IR observed in ACTIVE, we explored the potential effectiveness of ABL in historical trial populations, including those with higher placebo IRs than that in ACTIVE. This investigation assumed that the RRR observed in ACTIVE would be maintained in study populations with varying baseline risk and who were treated for periods longer than the 18 months of ABL treatment in ACTIVE. These assumptions were based on the apparent consistency of effect associated with ABL treatment over time [5, 18, 19] and across patient risk subgroups [14, 20].

We therefore calculated projected NNTs for ABL by applying the 86% RRR evident in ACTIVE to three historical populations, two of which had greater placebo IRs than did ACTIVE. With a 15% placebo IR, such as in FIT-1 [11], the NNT for ABL was projected to be 8, and with a placebo IR of 7%, such as in FREEDOM [10], the NNT for ABL was projected to be 17. For comparison, in FIT-1, the NNT for alendronate after 3 years of treatment was 14, and in FREEDOM, the NNT for denosumab after 3 years of treatment was 21. With a 4% placebo IR, such as that seen in FIT-2 and which is similar to the placebo IR observed in ACTIVE, the estimated NNT for ABL was 31. For comparison, in FIT-2, the NNT for alendronate was 60 after 4 years of treatment [12].

In a trial of up to 24 months of treatment with TPTD or placebo, the IR of new vertebral fracture among 448 women in the placebo group was 14%; among patients who received TPTD, the IR was 5% for 20 µg/d (*n* = 444 women) and 4% for 40 µg/d (*n* = 434 women), representing RRRs of 65 and 69%, respectively [21]. Based on the 9.3% ARR for TPTD 20 µg/d compared with PBO, Lewiecki et al. calculated the NNT for TPTD in that trial to be 11 [22]. The relationship between the NNT for TPTD (11, with 14% placebo-group IR) reported by Lewiecki and the NNT of 8 projected for ABL in a 15% IR population (as in FIT-1) is consistent with the relationship between the NNT of ABL and the NNT of TPTD observed in the ACTIVE population.

NNT calculations are highly dependent on baseline fracture risk and fracture IRs. A limitation of this analysis is that, for the historical comparisons, the RRR for vertebral fracture observed with ABL treatment during ACTIVE was assumed to be consistent in historical populations that included patients with varying levels of baseline risk, as well as varying study duration. Because the treatment effect with ABL observed in ACTIVE had a high degree of constancy across multiple timepoints during the 18-month treatment period and the 24-month alendronate monotherapy treatment period of ACTIVExtend [5, 18, 19], we assumed a similar consistent treatment effect across trials of varying study duration. Additionally, since the effects of ABL on fracture risk reduction appear to be independent of baseline risk factors and FRAX® fracture probability [14, 20], we assumed a consistent effect across trials with patients of varying baseline risk compared with ACTIVE. Despite these assumptions, however, results of the historical comparisons should be interpreted with caution.

## Conclusions

After 18 months of treatment in ACTIVE, the NNT for ABL was similar to that for TPTD for new vertebral fractures and lower than that for TPTD for nonvertebral, clinical, and major osteoporotic fractures. In historical populations with higher placebo IRs of vertebral fractures than were enrolled in ACTIVE, the projected NNT for ABL was lower than the calculated NNT using the ACTIVE trial population and was similar to the NNT for TPTD in the historical trial population with a similarly higher placebo IR. While such comparisons must be interpreted with caution, the NNT calculated for ABL was consistently lower than that for the active treatment arms of each historical trial analyzed, regardless of placebo IR. These data are useful for further evaluating ABL for the treatment of osteoporosis in postmenopausal women.
